# Late Adverse Health Outcomes and Quality of Life after curative radiotherapy + long-term ADT in Prostate Cancer Survivors: Comparison with men from the general population

**DOI:** 10.1016/j.ctro.2022.08.003

**Published:** 2022-08-06

**Authors:** Sophie D. Fosså, Alv A. Dahl, Tom Børge Johannesen, Ylva M. Gjelsvik, Anne Holck Storås, Tor Å. Myklebust

**Affiliations:** a Department of Oncology, Oslo University Hospital, Oslo, Norway; b Institute of Clinical Medicine, University of Oslo, Oslo, Norway; c Department of Oncology, Oslo University Hospital, Oslo, Norway; d Department of Registration, Cancer Registry of Norway, Oslo, Norway; e Department of Registration, Cancer Registry of Norway, Oslo, Norway; f Department of Oncology, Oslo University Hospital, Oslo, Norway; g Department of Research, Møre and Romsdal Hospital Trust, Ålesund, Norway

**Keywords:** Prostate cancer, Radiotherapy, Long-term Adverse Health Outcomes, Quality of Life, General population

## Abstract

**Background:**

Few studies have described the impact of urinary, bowel and sexual Adverse Health Outcomes (AHOs) on Quality of Life (QoL) in Prostate Cancer Survivors living for more than 5 years after curative radiotherapy (“long-term PCaSs”), and compared the findings with those in men from general population. Here we assess self-reported AHOs in such PCaSs focusing on the association between problem experience and QoL. The findings are compared to corresponding symptoms in age-similar men from the general population without a PCa diagnosis (Norms).

**Methods:**

Nine years (mean) after curative radiotherapy 1231 PCaSs and 3156 Norms completed the EPIC-26 questionnaire and the EORTC QLQ-C30 instrument. Domain Summary Scores (DSSs) for the urinary, bowel and sexual domains, the percentages of moderate/big dysfunctions and the proportions of overall problems were determined. Inter-cohort differences were interpreted based on cut-off values for published Minimal Clinically Important Differences (MCIDs). Multivariable linear regression models analyzed the associations between QoL and domain-related overall problems.

**Results:**

Only the inter-cohort differences regarding bowel and sexual DSSs exceeded the respective MCIDs. Among PCaSs 54% had at least one moderate/big problem (Norms: 30%). In PCaSs and Norms, QoL increased with decreasing urinary and bowel problems, For sexuality this association was weaker in Norms and was almost lacking in PCaSs. Multivariable-adjusted QoL was similar in PCaSs and Norms, with general health being the strongest covariate.

**Conclusions:**

During follow-up of long-term PCaSs health professionals should be aware of the survivors’ persisting moderate/big urinary, bowel or sexual problems associated with reduced QoL. In particular , alleviation of urinary and bowel problems can increase the men’s QoL.

## Highlights

• More than 5 years after radiotherapy for prostate cancer ) 54 % elderly patients report at least one moderate or big problem within the urinary, bowel or sexual domain. (Controls : 30%)
• Such problems reduce Quality of Life., which, however ,is similar in the two cohorts , the least difference observed within the sexual domain.

## 1 Introduction

Radiotherapy (RT) with or without adjuvant androgen deprivation treatment (ADT) of varying duration represents a curative treatment of non-metastatic prostate cancer (PCa), the survival rates dependent on the radiation dose. However, with rising target doses the risk of urinary, bowel and sexual Adverse Health Outcomes (AHOs) (“typical AHOs”) increases, with eventual negative impact on Quality of Life (QoL). New radiotherapy (RT) techniques such as Intensity- modulated radiotherapy (IMRT), Image Guided Radiotherapy, Volumetric Modulated Arc Therapy (VMAT) and new brachytherapy techniques are expected to reduce this risk [1].

Typical post-RT AHOs have in PCa survivors (PCaSs) been described in multiple studies [2], [3], [4], [5], [6], [7], [8], [9], [10], [11] but few reports, often from non-European institutions, have dealt with PCaSs living for more than five years after RT (“Long-term PCaSs”) [2], [3], [4], [5], [8], [9], [10], [11]. Moreover, the numbers of evaluated long.term PCaSs have often been limited (<200). On the background of PCa patients’ 10-year post-RT overall survival of ≥75 % [12] and the considerable between-country variability of self-reported AHOs [13] more and larger studies should deal with post-RT AHOs and related Quality of Life (QoL) in European long-term PCaSs, comparing the findings with corresponding symptoms in age-similar men from the general population (Norms).

The two co-primary aims of this nation-wide observational cohort study in long-term PCaSs are therefore.

1. To describe the severity of patient-reported typical AHOs after RT combined with ADT as well as the prevalence of rmoderate or big typical dysfunctions and related problems.

and

2. To assess the associations between overall urinary, bowel and sexual problems and QoL.

All findings in PCaSs are compared with corresponding observations in Norms.

## Patients and methods

### General

For each PCa patient diagnosed in the country the Cancer Registry of Norway (CRN) documents basic diagnosis- and treatment-related data, collects data on total and daily radiation doses and the number of daily fractions. Following the Norwegian guidelines from 2009 and 2015 (https://www.helsedirektoratet.no) curative RT for PCa implied a total dose of ≥70 Gy, applied by a 3-or 4- field conformal technique most often combined with 2–3 years of neoadjuvant ADT. Since 2011 IMRT was gradually used by the country’s nine radiotherapy units. MVAT was first introduced in 2017. The most frequent daily dose was 2 Gy. High-dose rate brachytherapy combined with external beam RT (HDR / EBRT) was also used at one hospital [14] and hypo-fractionated RT (HYPO-RT) [15] at another center. Margins of 10 mm to the rectum were viewed acceptable, with weekly verifications of the target volume.

### PCa survivors

Based on a previous study [16] we identified relapse-free 4306 PCaSs diagnosed from 2004 to 2015 who started curative RT to the prostate before 2017 and were ≤80 years old per August 1rst, 2021. These PCaSs were invited to complete a questionnaire presented to them on a specified Internet page.

### Norms

The CRN had randomly identified 10,843 men from the general population (2017–2019) without a PCa diagnosis but similarly aged as men with PCa. Totally 9509 of these men, aged within the age range of the study’s PCaSs, were invited to complete the same questionnaire as presented to the PCaSs, omitting PCa-related questions.

### The questionnaire

The questionnaire contained the Norwegian versions of EPIC-26 [17] and the EORTC QLQ-C30 instrument [18]. The current analyses disregard the hormonal domain of EPIC-26, but uses Question 13c (depression) and Question 13d (lack of energy) for descriptive purposes. The urinary, bowel and sexual Domain Summary Scores (DSSs) were calculated (https://medicine.umich.edu/), each DSS reflecting the severity of the domain’s AHOs, ranging from 0 (worst) to 100 (absent). Minimal Clinically Important Differences (MCIDs) assessed differences between DSSs [19]: Urinary incontinence: 6; Urinary obstruction/irritation: 5; Bowel 4; Sexual : 10. The percentage of non-valid EPIC-26 domains was ≤6 %, and <1 % of responders had no valid domain. Cronbach alpha was >0.75 for each of the valid DSSs.

Each domain in EPIC-26 covers aspects of functional impairment (“dysfunctions”). Within the bowel and sexual domain one additional question and question 5 of EPIC-26 assess overall urinary, bowel and sexual problems, ranked as “No problem”, “Very small problems”, “Small problems”, “Moderate problems“ and “Big problems”. Following Downing et al [5] we determined the proportions of men with the two worst response alternatives of each EPIC-26 item, briefly called “*substantial* dysfunction” or “*substantial* problem”.

Responses to Item 30 of the EORTC QLQ-C30 reflected QoL. The original scale of item 30 , ranging from 1 to 7, was transformed covering 0 (worst) to 100 ( best) points or was dichotomized: 1–4: (poor QoL) versus 5–7 (satisfactory QoL) [18]. Inter-cohort differences of >10 points were viewed as moderate”, contrasting “small” differences (≤10 points) [20]. Based on a previous study [21] we also included the following variables from C30: General health (Item 29, operationalized as Item 30), work capacity (Item 6), leisure activity (Item7), and social activity (Item 27) the latter three responses dichotomized: 1–2 (Not limited) versus 3–4 (Limited).

### Statistics

Standard descriptive methods were used, presenting means and corresponding standard deviations (SDs) of continuous variables, and absolute and relative frequencies of categorical variables. Due to considerable differences in the age distribution between PCaSs and Norms, descriptive statistics for Norms were age-adjusted, based on three age categories (<70, 70–<75, ≥75 years).

Following the principles laid out by causal inference theory it can be argued that sexual, bowel and urinary problems affect overall health, social function and other parts of everyday life [21] so that such factors may be considered to mediate the effect of the three AHO-related overall problems on QoL. The first linear regression model (Model 1) therefore assessed the associations between levels of QoL and the five degrees of sexual, bowel and urinary overall problems, only adjusting for age and level of education (<college vs ≥college) as confounders. Interaction terms between case-control status and all other covariates were included together with the before mentioned confounders. Model 2 also included general health, work capacity, leisure activity and social activity as covariates. Predicted levels of QoL for selected covariate patterns were obtained at the means of all other covariates. We used likelihood ratio tests to test the models including interaction effects with age group, but these were not close to significant. These added complexities were thus deemed unnecessary. Statistical significance: p < 0.05. SPSS version 26.0 and Stata version 17.0 were used.

### Ethics

The Regional Committee for Medical Research Ethics South-East approved this study (no.165867).

## 3 Results

With similar response rates in both groups 1,231 relapse-free PCaSs, and 3,156 Norms were finally evaluable (suppl. Fig. 1). Adjusting for age significantly more PCaSs than Norms reported poor general health, limited work capacity and problems with leisure or social activity (Table 1). Also depression and lack of energy were in PCaSs significantly increased. The target dose was >70 Gy in 90 % of the PCaSs, and about 60 % had undergone IMRT. HDR / EBRT had been applied in 140 PCaSs and HYPO-RT had been applied in 219 men (suppl. Table 1).

**Table 1:** PCaSs and Norms: Characteristics.

	**PCa Survivors** n: 1231 (%)	**Norms** n: 3156 (%)	
		Age-adjusted	Age-Unadjusted
**Demographics**			
**Age at Survey** All* 55 – 69.9 years 70 – 74.9 years ≥75 – 80 years	74.5 (4.3) 195 (16%) 405 (33%) 631 (51%)	73.4 (4.9) 500 (16%) 1038 (33%) 1618 (51%)	68.7 (5.9) 1646 (52%) 986 (31%) 524 (17%)
**Civil status** Married/living together Single	1010 (82%) 215 (18%)	2509 (80%) 620 (20%)	2507 (80%) 623 (20%)
**Education** <College College /University	580 (48%) 635 (52%)	785 (58%) 1332 (42%)	1689 (54%) 1434 (46%)
**EORTC QLQ- C30**			
**Limited Work capacity** No (Score 1–2) Yes (Score 3–4)	1032 (84%) 196 (16%)	2840 (91%) 287 (9%)	2854 (91%) 277 (9%)
**Limited Leisure activity** No (Score 1–2) Yes (Score 3–4)	1041 (85%) 189 (15%)	2818 (91%) 277 (9%)	2851 (92%) 258 (8%)
**Limited Social activity** No (Score 1–2) Yes (Score 3–4)	1022 (83%) 200 (16%)	2882 (92%) 237 (8%)	2910 (93%) 220 (7%)
**General health** All* Satisfactory (score 5–7) Poor(score 1–4)	71.2 (27.9) 888 (72%) 342 (28%)	81.2 (19.8) 2734 (87%) 410 (13%)	81.5 (19.6) 2761 (88%) 387 (12%)
**EPIC-26**			
**Depression** All* No/Very small/Small Moderate/Big	86.5 (23.7) 1114 (93%) 68 (7%)	91.7 (18.4) 2910 (97%) 91 (3%)	91.3 (18.8) 2958 (97%) 94 (3%)
**Lack of Energy** All* No/Very small/Small Moderate/Big	68.9 (31.0) 957 (86%) 137 (14%)	81.2 (24.7) 2797 (92%) 226 (8%)	81.5 (24.5) 2848 (94%) 226 (6%)

*Mean (Standard Deviation).

In spite of statistically significant differences between the age-adjusted DSSs (p < 0.01), only the inter-cohort differences of the bowel and the sexual DSSs exceeded the respective MCIDs (Table2). Compared to all Norms the sexual DSS in the group of PCaSs was almost halved (31.9 vs 55.4). Further, age- related differences of sexual DSSs were in PCaSs larger than in Norms, without similar findings for the urinary or bowel DSSs. The DSSs in PCaSs who had IMRT or HYPO-RT were similar to figures after RT without IMRT (suppl. Table 3). PCaSs who had undergone HDR / EBRT had the most favorable DSSs.

**Table 2:** A. Domain Summary Scores (DSSs) and mean Overall problems (EPIC-26); B: Quality of Life (QLQ-C30).

A: EPIC-26	**PCaSs**				**Norms**				
					**Age-adjusted**				**Age-Un-adjusted**
***DSS/ overall urinary problems***	**<70y**	**70**–**74.9y**	**≥75y**	**Total**	**<70y**	**70**–**74.9y**	**≥75y**	**Total**	
Urinary incontinenceDSS (SD)*	88.8 (19.9)	88.1 (18.7)	86.1 (20.7)	87.2 (20.0)	93.5 (12.7)	90.9 (15.4)	89.6 (17.6)	90.7 (16.2)	92.0 (14.5)
Urinary Irrit./Obstr.DSS (SD)*	82.7 (16.8)	82.1 (17.0)	82.1 (14.4)	82.2 (16.9)	87.7 (14.0)	85.1 (14.0)	84.4 (15.1)	85.5 (14.6)	86.5 (14.2)
Overall urinary problem Mean (SD)*	73.8 (28.7)	74.7 (26.4)	73.3 (29.0)	73.8 (28.1)	82.9 (23.1)	78.7 (25.2)	78.8 (25.8)	79.4 (25.2)	80.9 (24.3)
BowelDSS (SD)	82.8 (19.9)	82.4 (20.0)	84.0 (18.0)	83.3 (19.1)	93.5 (11.9)	93.9 (18.6)	92.2 (13.0)	93.0 (12.5)	93.4 (12.1)
SexualityDSS (SD)*	43.7 (27.2)	33.7 (25.8)	27.0 (22.8)	31.9 (25.3)	73.3 (25.6)	58.8 (28.1)	47.5 (28.9)	55.4 (29.6)	64.6 (28.6)
**B: QLQ-C30**									
Quality of Life (Item 30)Mean (SD)*	74.4 (24.1)	73.5 (23.5)	73.0 (22.7)	73.4 (23.2)	83.2 (19.9)	84.5 (18.6)	82.6 (20.3)	83.3 (19.7)	83.5 (19.6)
Satisfactory (5-7) Poor (1-4)	148 (76%) 47(24%)	303 (75%) 102(25%)	483 (77%) 147(23%)	934(76%) 296 (24%)	443 (89 %) 56 (11 %)	941 (91 %) 93 (9 %)	1411 (87 %) 207 (13 %)	2795 (89 %) 356 (11 %)	2811 (89 %) 339 (11 %)

*Mean (Standard Deviation).

**Fig. 1:**
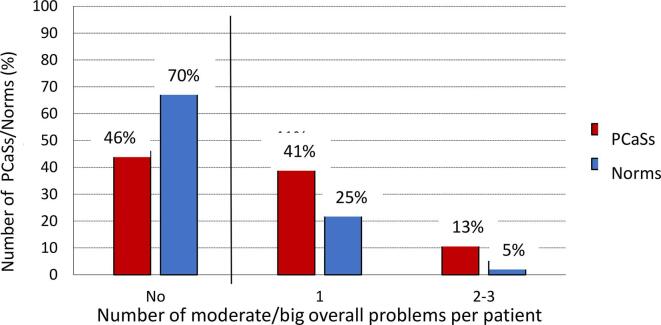
Age-adjusted percentages of PCaSs and Norms with no, 1 or 2–3 moderate or big overall problems within the urinary, bowel or sexual domains. (P < 0,01 for all inter-group differences).

**Fig. 2:**
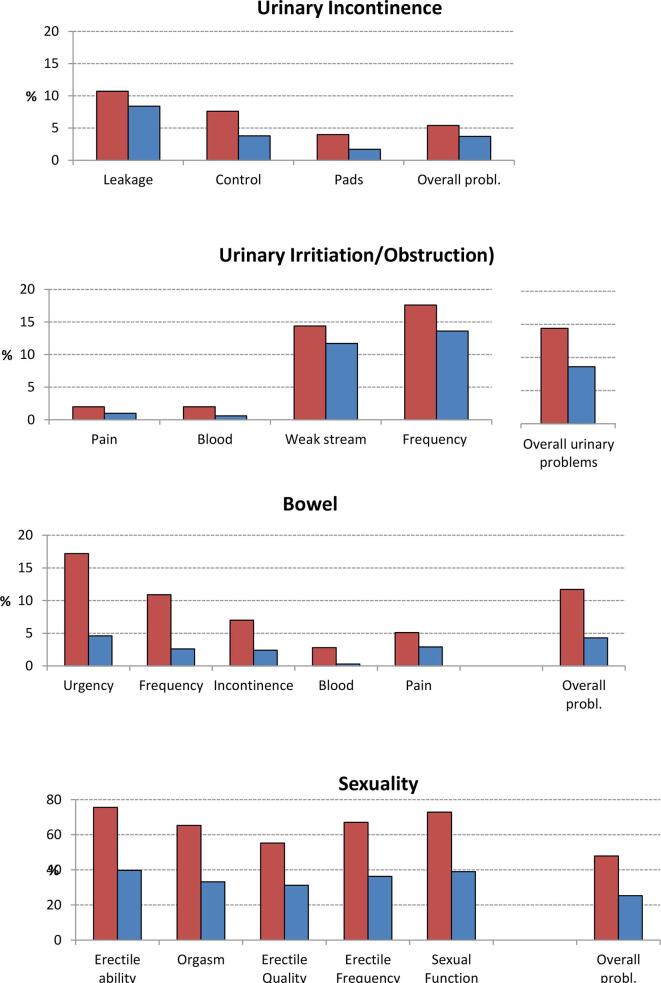
Age-adjusted percentages of PCaSs  and Norms  with domain-specific moderate or big AHOs or overall problems (p < 0,01 for all inter-group differences except for pain [p:0.02]).

Forty-six percent of the PCaSs did not record any substantial problem compared to 70 % of the Norms (Fig. 1). All proportions of PCaSs with substantial dysfunctions or overall problems exceeded the corresponding percentages among Norms (Fig. 2, suppl. Table 2). About 15 % of the PCaSs reported substantial urinary or bowel problems, while the prevalence of sexual problems was almost 50 %. Further, compared to the Norms the urinary and sexual problems were almost doubled in the PCaSs along with a nearly threefold increase of bowel problems. Notably, substantially reduced sexual function (EPIC item no 11) was described by 73 % of the PCaSs, but only 48 % reported substantial overall sexual problems. The corresponding figures among Norms were 40 % and 25 %.

Table 3 documents the independent associations between QoL and the rankings of urinary, bowel and sexual problems. According to the regression coefficients in Model1 the associations between QoL and urinary or bowel problems are in the PCASs much stronger than between QoL and sexual problems. After adjusting for the mediating variables and covariates (Model 2), the reduction of urinary and bowel problems remained significantly associated with increasing Qol levels, though weaker than in Model1. In particular, the association between sexual problems and QoL became less evident. Both in the PCaSs and the Norms general health was the dominating predictor of QoL. Importantly, the mean QoL levels based on Model 2 were similar in PCaSs and Norms (PCaSs: 79 [range: 78–80]; Norms: 81 [range: 81–82]).

**Table 3:** Multivariate regression analyses with QoL as dependent variable.

	**Model 1**		**Model 2**	
**Variables**	**PCaSs**	**Norms**	**PCaSs**	**Norms**
Age (ref: <70 years) 70-74 75+	2.6 (1.3,4.0)^1^ 2.1 (0.6,3.6)	2.6 (1.3,4.0) 2.1 (0.6,3.6)	1.4 (0.5,2.4) 1.8 (0.7,2.9)	1.4 (0.5,2.4) 1.8 (0.7,2.9)
Education (ref: <College) College/University	2.6 (1.5,3.7)	2.6 (1.5,3.7)	0.6 (-0.2,1.4)	0.6 (-02,1.4)
General health (ref: Satisfactory) Poor	NR	NR	−28.0 (-30.1,-26.0)	−29.2 (-31.0,27.3)
Limited leisure activity (ref: No) Yes	NR^2^	NR	−9.9 (-12.1,-6.7)	−2.9 (-5.6,-0.1)
Limited work capacity (ref: No) Yes	NR	NR	−1.9 (-5.0,1.1)	−6.5 (-9.1,-3.9)
Limited social activity (ref: No) Yes	NR	NR	−6.7 (-9.4,-4.0)	−10.0 (-12.4,-7,6)
Urinary problem (ref: Big) Moderate Small Very small None	11.7 (5.1,18,3) 14.1 (7.5,20.6) 21.5 (15.2,27.7) 26.3 (20.0,32.6)	4.0 (-2.3,10.4) 11.5 (5.3,17.7) 15.0 (9.0,21.0) 18.7 (12.7,24.7)	4.10 (-0.7,8.9) 5.6 (0.9,10.4) 7.5 (2.9,12.0) 11.4 (6.8,16.0)	5.4 (0.8,10.0) 6.0 (1.5,10.4) 7.2 (2.9,11.6) 10.8 (6.4,15.1)
Bowel problem (ref: Big) Moderate Small Very small None	14.9 (8.0,21.8) 21.6 (14.9,28.3) 23.4 (17.0,29.9) 28.8 (22.4,35.2)	4.7 (-4.0,13.4) 10.3 (1.9,18.8) 16.3 (8.1,24.4) 22.7 (14.6,30.8)	2.5 (-2.5,7.5) 3.8 (-1.0,8.7) 3.6 (-1.1,8.4) 7.1 (2.5,11.8)	1.1 (-5.4,7.6) 4.1 (-2.1,10.4) 5.6 (-0.5,11.6) 9.52 (3.5,15.5)
Sexual problem Moderate Small Very small None	7.0 (3.9,10.0) 7.0 (3.9,10.1) 8.0 (4.8,11.2) 8.6 (5.2,12.0)	2.8 (-0.3,5.8) 5.0 (2.1,8.0) 7.5 (4.7,10.4) 8.8 (6.1,11.6)	2.9 (0.7,5.1) 2.8 (0.6,5.1) 3.8 (1.5,6.1) 3.7 (1.3,6.2)	−1.1 (-3.4,1.1) −0.3 (-2.4,1.9) 1.3 (-0.7,3.4) 2.7 (0.7,4.7)

^1^ Non-standardized regression coefficients (95% confidence interval); ^2^Not Relevant.

**Fig. 3:**
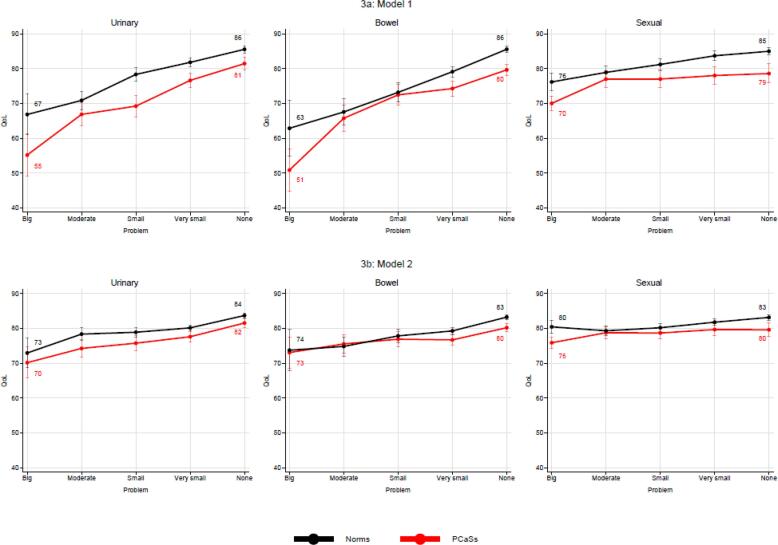
Associations between QoL and overall problems in PCaSs and Norms based on Model 1 (3a) and Model 2 (3b). (The numbers in each panel indicate the level of QoL for PCaSs and Norms associated with the respective ranking of Overall problems).

Fig. 3 visualizes the above findings. In PCaSs and Norms an almost linear increase of QoL is documented along with reduction of bowel and urinary problems (Fig. 3a). A much less steep improvement of QoL emerged in the Norms along with reduced sexual problems. In the PCaSs the QoL levels remained almost unchanged in men reporting Moderate, Small, Very small or None sexual problems. Reduction of urinary and bowel problems from Big to None increased QoL in PCaSs by nearly 30 points with less QoL improvement along with reduction of sexual problems (9 points). The corresponding QoL differences were generally lower in Norms. Controlling for mediating variables (Fig. 3b) reduced the absolute impact of urinary, bowel and sexual problems, but supported the effect of decreasing urinary and bowel problems on Qol improvement. The weaker impact of reducing sexual problems was confirmed, in particular for PCaSs. For each step of problem experience the QoL differences between PCaSs and Norms were small.

## 4 Discussion

In this nation-wide survey, 54 % of long-term PCaSs but only 30 % of Norms reported at least one moderate or big post-RT urinary, bowel or sexual overall problem. An almost linear QoL increase emerged in PCaSs and Norms along with decreasing overall urinary and bowel problems. The corresponding association regarding overall sexual problems was weaker in Norms and was almost lacking in PCaSs. Adjusted QoL was similar in PCaSs and Norms.

The DSSs and the means of overall problems in our PCaSs are comparable to published figures from EPIC-based surveys performed in PCa survivors >5 years after RT (Table 4), and our findings in Norms correspond well with those in age-similar men from the general population in Northern Ireland [22]. None of the relevant publications in PCaSs provide data on the duration of ADT, though a negative long-term impact of adjuvant ADT on sexual DSS has been indicated by Downing et al [5] and Hoffman et al [3]. Notably, the most favorable sexual DSS combined with a relatively high prevalence rate of preserved sexual function (64 %) emerged in Donovan et al’s [10] patients. Compared to our PCaSs the UK patients were, however, younger and had undergone ADT for maximum six months (Personal communication, M. Mason). Adjuvant ADT for 2–3 years has, on the other hand been “clinical practice” in Norway before 2017. High age, long-lasting ADT and insufficient recovery from hypogonadism represent the most probable explanation for the substantial erectile dysfunction in 75 % of our PCaSs and the >20 points inter-cohort difference of the sexual DSSs. RT-induced atherosclerosis of the pudental vessels [23] and scattered testicular irradiation [24] may have contributed to the development of late hypogonadism and of the low sexual DSS.

**Table 4:** Published observations on DSSs and/or overall problems >5 years after curative radiotherapy of prostate cancer.

**First author (ref. nr)**	**Observation time/#PCaSs**	**Urinary incontinence**	**Urinary irrit./obstr.**	**Overall urinary probl./funct.**	**Bowel**	**Sexual**
Taylor (8)	10 yrs n: 110			84 (2)	84 (2.7)^2^	28 (5.8)^2^
Resnick (2)	15 yrs n: 491^4^			∼78^3^	∼78	∼17
Miller (9)	6.2 yrs n: 147	86 (81–90)1	84 (80–87)		84 (81–86)	35 (29–41)
Punnen (7)	5–10 yrs n: 158			∼88	∼85	∼28
Pinkawa (6)	9–12 yrs n: 191				∼85	∼9
Donovan (10)	6 yrs n: ∼450	89 (14)^2^	93 (8)	90 (11)	91 (11)	41 (25)
Current study	9 yrs n: 1231	87 (20)	82 (17)	74 (28)	83 (19)	32 (25)

^1^Mean (range); ^2^Mean (SEM/ Standard deviation); ^3^Figure extracted from a relevant graph; ^4^Number of patients at start of the longitudinal study.

Long-lasting hypogonadism rises the risk of physical and mental morbidity [25], and explains the increased prevalence of depression and energy loss in our PCaSs. This finding, possibly more than sexual dysfunction itself, warrants the consideration of testosterone replacement therapy in long-term tumor-free PCaSs with severe symptoms and low serum testosterone [26], and supports today’s shorter ADT duration, whenever possible.

More PCaSs than Norms reported substantial overall urinary problems (14 % versus 9 %; p: <0.01), mostly due to increased micturition frequency and weak stream, neither uncommon in our Norms. Post-RT pelvic and bladder wall fibrosis [27] adds to the age-related micturition dysfunction reported by Norms.

Our bowel DSS is lower than Bergengren et al’s [11] Epic-26-based nation-wide results. Our figures are also considerably below Donovan et al’s figures in patients initially included in the ProtecT trial [10]. The latter difference is possibly related to the common outcome differences between individuals selected to participate in trials and those included in population-based surveys [28]. Further, older age of our PCaSs, larger primary tumors and increasing post-RT fibrosis along with expanded time since RT contributes the differences between our and the UK findings.

We could not confirm data on reduced post-RT toxicity using IMRT [29], but document slightly more favorable findings after the use of HDR / EBRT. Gradual increase of the total dose for EBRT may be one explanation for this disappointing finding together with the acceptance of a 10 mm posterior margin. In agreement with published findings Hypo-RT did not increase the severity of typical AHOs [30].

EPIC-26 is internationally recommended for assessment of post-treatment AHOs in PCaSs [31]. The instrument is often referred to as a QoL instrument, though the questionnaire does not cover items important for a PCaS’ generic QoL such as work capacity and leisure or social activities [21]. In some studies EPIC-26 has therefore been supplemented by a generic Qol instrument [3], [4], [5] such as the EORTC QLQ-C30 in the current study [18]. This approach increases the understanding of the associations between PCaSs’ QoL and the PCa- typical AHOs. Not surprisingly, our data indicate that the stepwise reduction of urinary bowel and problems increases QoL in PCaSs and Norms. As also discussed by others [2], [4], [5], [6] for the sexual domain this association was in our PCaSs only moderate or weak: Only about two thirds of our PCaSs reporting substantial erectile dysfunction also described big or moderate sexual function problems. We can only speculate about an explanation of the limited association between sexual problems and QoL in PCaSs. In contrast to the age-related gradual decrease of sexual function in the Norms, PCaSs experience loss of sexual functions soon after ADT start, not rarely with insufficient recovery after 2–3 years of ADT. Response shift [32] and satisfactory social and leisure activities [21] may have reduced the survivor’s view on the importance of sexual function for his QoL. Further, important emotional and relational issues of sexuality, stronly associated with elderly men's QOL, are not covered by EPIC-26.

In agreement with other studies [3], [4], [5] the inter-cohort differences of QoL were small in Model 2, and we document the highly significant association between QoL and general health. Without access of relevant pre-treatment characteristics, the causal influence of RT on our PCaCs’ general health cannot be quantitated. On the other hand, RT, combined with long-lasting ADT has most probably contributed to the excess rates of energy loss and depression in PCaSs, these conditions impacting on a PCaS’ experience of poor general health.

### Limitations and strengths

Our registry-based study has several limitations. Only about one third of the invited men participated in the survey, the low compliance possibly related to the men’s high age, reduced health and lack of Internet competence. Further, the RT techniques used could only be broadly described, disabling to study more detailed correlations between RT and AHOs. Neither do we have any information on therapeutic procedures performed to reduce severe problems in individual PCaSs. As detailed data on comorbidity were lacking , Item 29 of the C30 questionnaire served as a measure of general health, while only Item 30 reflected QoL thus slightly deviating from the recommended operationalization [18]. Further, we collected data from only one Northern-European country. Inter-country culture-dependent variations of self-reported urinary symptoms and, in particular, of sexuality among PCaSs and in men from the general population should not be ignored [13], [33]. Finally, our PCaSs were relatively old (mean age 74 years), and different results, not at least regarding sexual problems, are to be expected in younger men.

The large sample size of populations-based cohorts and the long observation time of the PCaSs represent the study’s advantages. The real-world design minimizes selection bias which must be considered when PCaSs from trials or single institutions are evaluated [28].

As far as we know, this is the first European report which compares post-RT long-term urinary, bowel and sexual AHOs between PCaSs and men from the general population and describes the associations between related problems and QoL.

## 5 Conclusion

About 10–15 % of long-term PCaSs suffer from post-RT big or moderate urinary or bowel problems, with sexual problems in 50 % of them. Such problems are two-to threefold increased compared to age-similar men from the general population, and they are inversely associated with the men’s QoL. Improvement of post-RT QoL can be expected by therapeutic tasks which alleviate these problems, in particular within the urinary and bowel domain. Overall, PCa patients can during pre-treatment counseling be informed that their long-term QoL after RT most probably will be similar to that of non-irradiated age-comparable peers. Future studies should address whether modern RT-techniques and today’s reduced ADT duration decrease the prevalence of moderate/big long-term post-RT problems thereby increasing QoL.

## Authors’ contribution

Fosså SD / Myklebust TAA: Principal co-investigators, manuscript writing, statistical analyses.

Johannessen TB / Gjelsvik Y: Data provision, comments to and interpretation of the findings.

Dahl AA / Storås AH: Comments to and interpretation of the findings.

## Funding

The Movember Foundation in partnership with the Cancer Registry of Norway, Norwegian Prostate Cancer Association.

## Declaration of Competing Interest

The authors declare that they have no known competing financial interests or personal relationships that could have appeared to influence the work reported in this paper.
