# Supplementary material for: Late Adverse Health Outcomes and Quality of Life after curative radiotherapy + long-term ADT in Prostate Cancer Survivors: Comparison with men from the general population
Source: Clin Transl Radiat Oncol. 2022 Aug 6;37:78–84. doi: 10.1016/j.ctro.2022.08.003 (PMC9450064; doi:10.1016/j.ctro.2022.08.003)
Supplement: Supplementary data 1 [file mmc1.docx]

**Suppl. Figure 1: Flow chart**

Evaluable

n: 1231

n: 3156

n: 209 (0.6%)

No valid domains

Excluded

n: 62 (0.4%)

No valid domains

n: 183

Current hormone therapy

n: 1476

(34%)

Responders

n: 3365

(35%)

**Norms**

N

9509*

Invited

**PCaSs**

4306

N

4306

***Number of invited men within the age range of the invited 10,843 PCaSs**

**(Cfr text)**
