# Supplementary material for: Late Adverse Health Outcomes and Quality of Life after curative radiotherapy + long-term ADT in Prostate Cancer Survivors: Comparison with men from the general population
Source: Clin Transl Radiat Oncol. 2022 Aug 6;37:78–84. doi: 10.1016/j.ctro.2022.08.003 (PMC9450064; doi:10.1016/j.ctro.2022.08.003)
Supplement: Supplementary data 2 [file mmc2.docx]

**Suppl Table 1 :Radiotherapy and medical data in PCaSs**

|  | **PCa survivors**  n: 1231 |
| --- | --- |
| **Age at RT(years)**  ALL  <70 at RT (n:195)  70-<75 at RT ( n:405)  ≥75 at RT (n:631) | 65.5 (5.0)*  58.2 (3.9)  64.9 (9.2)  68.5 (3.2) |
| **Years since RT**  Mean ( SD)  Range  5 - <7  7 - 10  ≥10 | 9.0 (2.7)  5-15  369 (30%)  450 (37%)  412 (34%) |
| **Technique**  *No IMRT*  *IMRT*  **Target doses**  EBRT  *2 Gy daily*  70 - <74  74 - <78  78  *80 (*Hypofractionated)  High-dose rate brachytherapy/EBRT  102 Gy | *463(42%)*  *628(58%)*  144 (12%)  420 (34%)  308 (25%)  219 (18%)  140 (11%) |
| **Risk group**  1  2  3  Missing | 99 (8%)  454 (37%)  647 (52%)  31 (3%) |
| **T-category**  T1 – T2  T3 – T4  Missing | 721 (59%)  336 (27%)  174 (14%) |

*Mean (Standard Deviation)
