# Supplementary material for: Late Adverse Health Outcomes and Quality of Life after curative radiotherapy + long-term ADT in Prostate Cancer Survivors: Comparison with men from the general population
Source: Clin Transl Radiat Oncol. 2022 Aug 6;37:78–84. doi: 10.1016/j.ctro.2022.08.003 (PMC9450064; doi:10.1016/j.ctro.2022.08.003)
Supplement: Supplementary data 3 [file mmc3.docx]

**Suppl. Table 2: Age-adjusted Percentages of PCaSs and Norms with moderate or big dysfunction/problems**

| **A: Urinary Incontinence** | **PCaSs** | | | | **Norms** | | | | |
| --- | --- | --- | --- | --- | --- | --- | --- | --- | --- |
|  |  | | | | **Age-adjusted** | | | | **Crude** |
|  | **<70y** | **70**-<75y | **≥75y** | **Total** | **<70y** | **70-<75y** | **≥75y** | **Total** | **Total** |
| Leakage  Q1 | 9.2%^1^ | 10.4% | 11.9% | 87.2 (28.5)^1^  10.7% | 5.3% | 7.6% | 9.9% | 89.6 (25.6)  8.4% | 91.5 (23.3)^1^  6.7% |
| Control  Q2 | 8.2% | 6.9% | 8.3% | 82.6 (22.6)  7.6%) | 2.4% | 3.6% | 4.4% | 86.4 (19.5)  3.8% | 88.0 (18.4)  3.1% |
| Pads  Q3 | 3.6% | 3.7% | 5.1% | 93.3 (18.8)  4% | 0.7% | 1.6% | 2.1% | 97.3 (12.4)  1.7% | 98.2 (10.2)  1.2% |
| Overall.Incont.  Problem  Q4a | 4.7% | 5.6% | 5.6% | 85.5 (21.8)  5.4% | 2.2% | 3.1% | 4.6% | 89.2 (19.6)  3.7% | 90.5 (18.6)  2.9% |

^1^ Item mean (Standard deviation)

| **B: Urinary Irritation/ Obstruction** | **PCaSs** | | | | **Norms** | | | | |
| --- | --- | --- | --- | --- | --- | --- | --- | --- | --- |
|  |  | | | | **Age-adjusted** | | | | **Crude** |
|  | **<70y** | **70-<75y** | **≥75y** | **Total** | **<70y** | **70-<75y** | **≥75y** | **Total** | **Total** |
| Pain  Q4b | 2.1% | 2.1% | 1.9% | 95.1 (14.6)^1^  2.0% | 1.1% | 1.0% | 1.1% | 96.7 (12.1)  1% | 97.0 (11.8)^1^  1.3% |
| Blood  Q4c | 2.1% | 2.1% | 1.9% | 95.1 (14.6)  2.0% | 0.4% | 0.2% | 0.8% | 98.7 (8.8)  0.6% | 99.0 (7.8)  0.4% |
| Weak stream  Q4d | 11.1% | 14.7% | 15.4% | 71.1 (23.4)  14.4% | 9.2% | 11.6% | 12.7% | 74.1 (26.7)  11.7% | 76.2 (26.2)  10.5% |
| Frequency  Q4e | 15.3% | 16.6% | 19.3% | 67.5 (30.2)  17.6% | 12.8% | 13.3% | 14.1% | 72.5 (28.0)  13.6% | 74.1 (27.8)  13.2% |
|  |  |  |  |  |  |  |  |  |  |
| **Overall urinary probl.**  **Q5** | 15% | 11.9% | 15.8% | 73.8 (28.1)  14.4% | 6.4% | 9.1% | 9% | 79.4 (25.2)  8.6% | 80.9 (24.3)  7.7% |

^1^ Item mean (Standard deviation)

| **C: Bowel Domain** | **PCaSs** | | | | **Norms** | | | | |
| --- | --- | --- | --- | --- | --- | --- | --- | --- | --- |
|  |  | | | | **Age- adjusted** | | | | **Crude** |
|  | **<70y** | **70-<75** | **≥75y** | **Total** | **<70y** | **70-<75y** | **≥75y** | **Total** | **Total** |
| Urgency  Q6a | 19.2% | 18.6% | 15.6% | 73.1 (30.2)^1^  17.2% | 4.1% | 3.1% | 5.8% | 89.3 (21.1)  4.6% | 90.2 (20.2)^1^  4% |
| Frequency  Q6b | 10.4% | 12.4% | 10.0% | 79.6 (27.4)  10.9% | 2.1% | 2.6% | 2.7% | 92.2 (17.5)  2.6% | 92.5 (17.2)  2.3% |
| Incontinence  Q6c | 4.1% | 9.1% | 6.6% | 86.7 (24.8)  7.0% | 0.9% | 1.3% | 3.5% | 95.8 (15.2)  2.4% | 96.7 (12.8)  1.4% |
| Blood  Q6d | 3.1% | 2.1% | 3.3% | 93.7 (17.5)  2.8% | 1.3% | 0.6% | 2.7% | 98.9 (6.6)  0.3% | 98.7 (7.7)  0.5% |
| Pain  Q6e | 5.2% | 6.2% | 4.3% | 89.2 (21.9)  5.1% | 2.6% | 3.1% | 2.9% | 93.5 (17.0)  2.9% | 93.3 (17.1)  2.8% |
| Overall problems  Q7 | 10.4% | 13.7% | 10.9% | 77.3 (27.6)  11.7% | 3.4% | 3.9% | 4.8% | 88.2 (20.7)  4.3% | 89.3 (19.1)  3.8% |

^1^ Item mean (Standard deviation)

| **D: Sexual**  **Domain** | **PCaSs** | | | | **Norms** | | | | |
| --- | --- | --- | --- | --- | --- | --- | --- | --- | --- |
|  |  | | | | **Age-adjusted** | | | | **Crude** |
|  | **<70y** | **70-<75y** | **≥75y** | **Total** | **<70y** | **70-<75** | **≥75y** | **Total** | **Total** |
| Erect.Ability  Q8a | 59.6% | 72.7% | 82.4% | 22.3 (27.8)^1^  75.6% | 18.3% | 34.9% | 49.7% | 47.8 (30.6)  39.7% | 56.9 (30.6)^1^  28.6% |
| Orgasm.Ability  Q8b | 45.1% | 61.3% | 74.4% | 28.9 (28.7)  65.3% | 13.0% | 26.9% | 43.7% | 51.4 (30.9)  33.2% | 61.2 (32.6)  22.3% |
| Erect.Quality  Q9 | 31.6% | 52.3% | 64.5% | 43.7 (36.2)  55.2% | 11.5% | 25.2% | 41.4% | 67.3 (36.7)  31.2% | 76.9 (32.6)  20.7% |
| Erect. Frequency  Q10 | 49.2% | 64.3% | 75.3% | 29.2 (34.7)  67.4% | 16.5% | 31.5% | 45.8% | 56.0 (39.2)  36.3% | 66.6 (37.0  25.9% |
| Sex. Function  Q11 | 55.4% | 69.0% | 81.0% | 24.2 (27.0)  72.9% | 18.5% | 34.1 | 48.9% | 47.1 (32.4)  39.0% | 57.1 (32.0)  28.3% |
| Overall Sex.Problems  Q12 | 50.0% | 48.5% | 46.8% | 43.1 (35.2)  47.9% | 15.3% | 23.2 | 29.8% | 63.6 (33.6)  25.3% | 69.1 (32.3)  20.1% |

^1^ Item mean (Standard deviation)
