# Supplementary material for: Late Adverse Health Outcomes and Quality of Life after curative radiotherapy + long-term ADT in Prostate Cancer Survivors: Comparison with men from the general population
Source: Clin Transl Radiat Oncol. 2022 Aug 6;37:78–84. doi: 10.1016/j.ctro.2022.08.003 (PMC9450064; doi:10.1016/j.ctro.2022.08.003)
Supplement: Supplementary data 4 [file mmc4.docx]

**Suppl. Table 3 : Radiotherapy technique: Domain Summary Scores and % Moderate/Big problems**

|  | **EBRT only** | | **HDR /EBRT**  **n: 140** |
| --- | --- | --- | --- |
| **DSS/Mean (SD)**  **%Mod./Big problems** | **No IMRT**  **n: 463**  ***n: 149***^1^ | **IMRT**  **n: 628**  ***n: 70*** |  |
| Urinary Incontinence | 86.5 (20.7)^2^  *87.7 (20.3)* | 87.1 (19.7)  *89.8 (16.4)* | 90.0 (18.5) |
| Urinary Irrit./Obstr. | 81.5 (17.2)  *81.2 (17.9)* | 81.8 (17.2)  *84.4 (28.3)* | 85.7 (14.3) |
| Overall Urinary Probl. | 72.0 (28.2)  *74.9 (28.4)* | 73.5 (28.5)  *75.4 (28.3)* | 80.5 (25.0) |
| Mod./Big problem | 14.7%^3^  *14.9%* | 15.6%  *7.1%^4^* | 8.8% |
| Bowel | 83.1 (18.4)  *85.9 (17.4)* | 82.1 (20.2)  *81.5 (20.5)* | 88.9 (14.0) |
| Mod./Big problem | 13.3%  *11.4%* | 12.2%  *10.0%* | 6.5% |
| Sexuality | 30.1 (23.5)  *34.2 (26.4)* | 31.2 (25.7)  *32.4 (28.3)* | 40.8 (27.0) |
| Mod./Big Problem | 48.3%  *50.7%* | 50.5%  *50.5%* | 38.1% |

^1^PCaSs with hypo-fractionated RT; ^2^Mean (Standard Deviation); ^3^Moderate/Big problems;

^4^Based on 5 PCaSs
